# Supplementary material for: LOXL2 in Cancer: A Two-Decade Perspective
Source: Int J Mol Sci. 2023 Sep 21;24(18):14405. doi: 10.3390/ijms241814405 (PMC10532419; doi:10.3390/ijms241814405)
Supplement: Supplementary file 1 [file ijms-24-14405-s001.zip › Figure S1.pptx]

## Slide 1
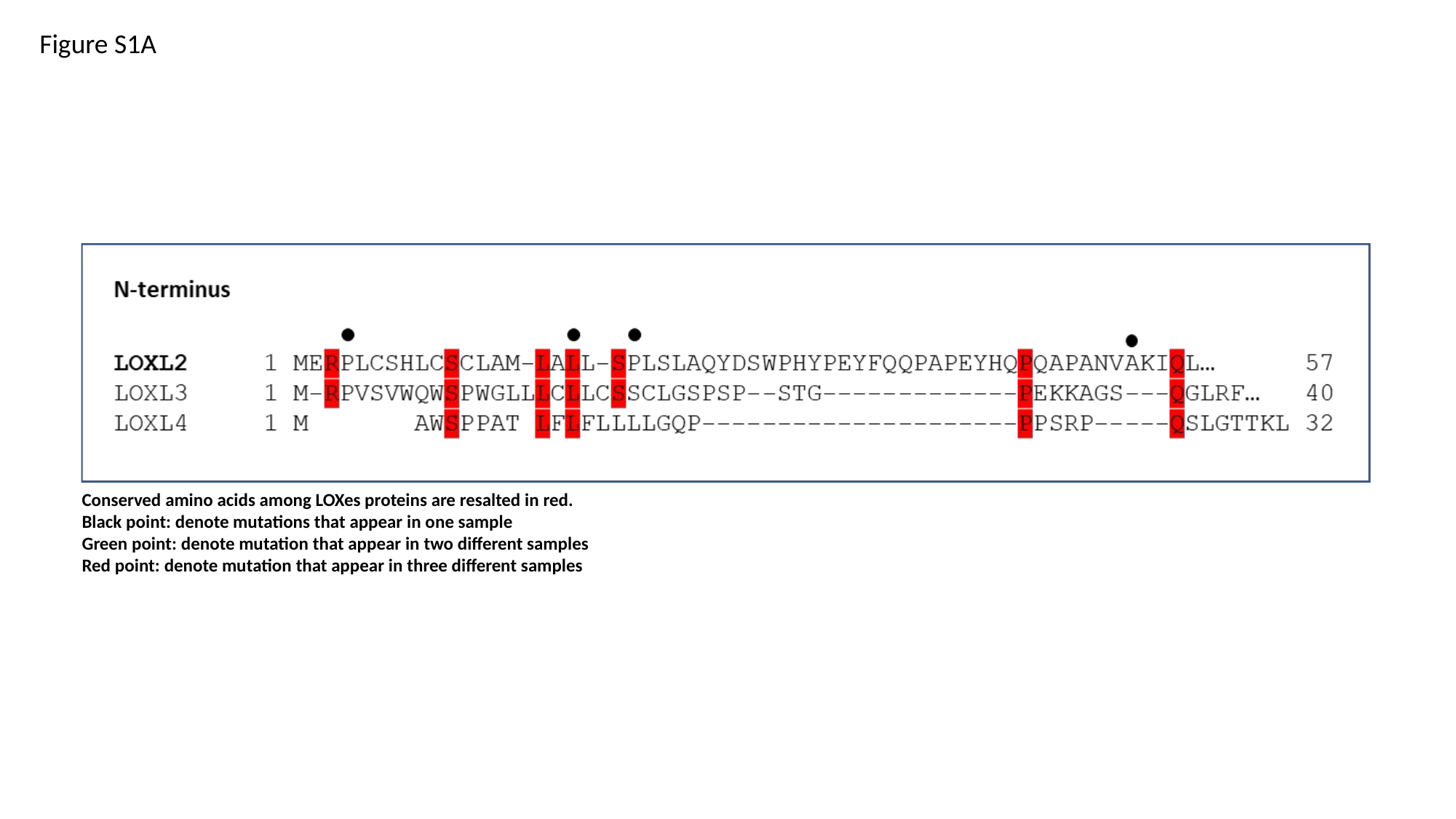

Figure S1A
Conserved amino acids among LOXes proteins are resalted in red.
Black point: denote mutations that appear in one sample
Green point: denote mutation that appear in two different samples
Red point: denote mutation that appear in three different samples

## Slide 2
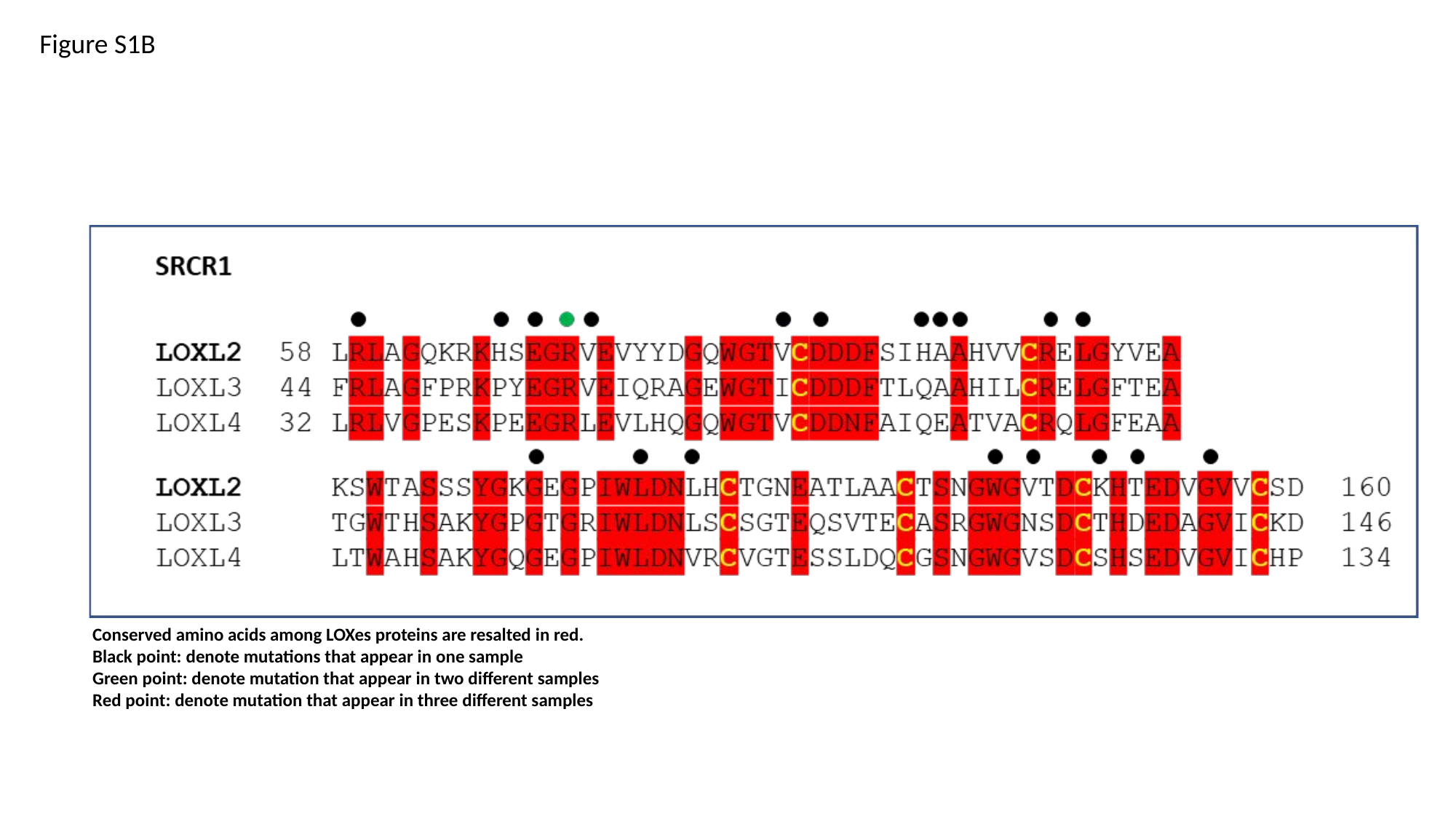

Figure S1B
Conserved amino acids among LOXes proteins are resalted in red.
Black point: denote mutations that appear in one sample
Green point: denote mutation that appear in two different samples
Red point: denote mutation that appear in three different samples

## Slide 3
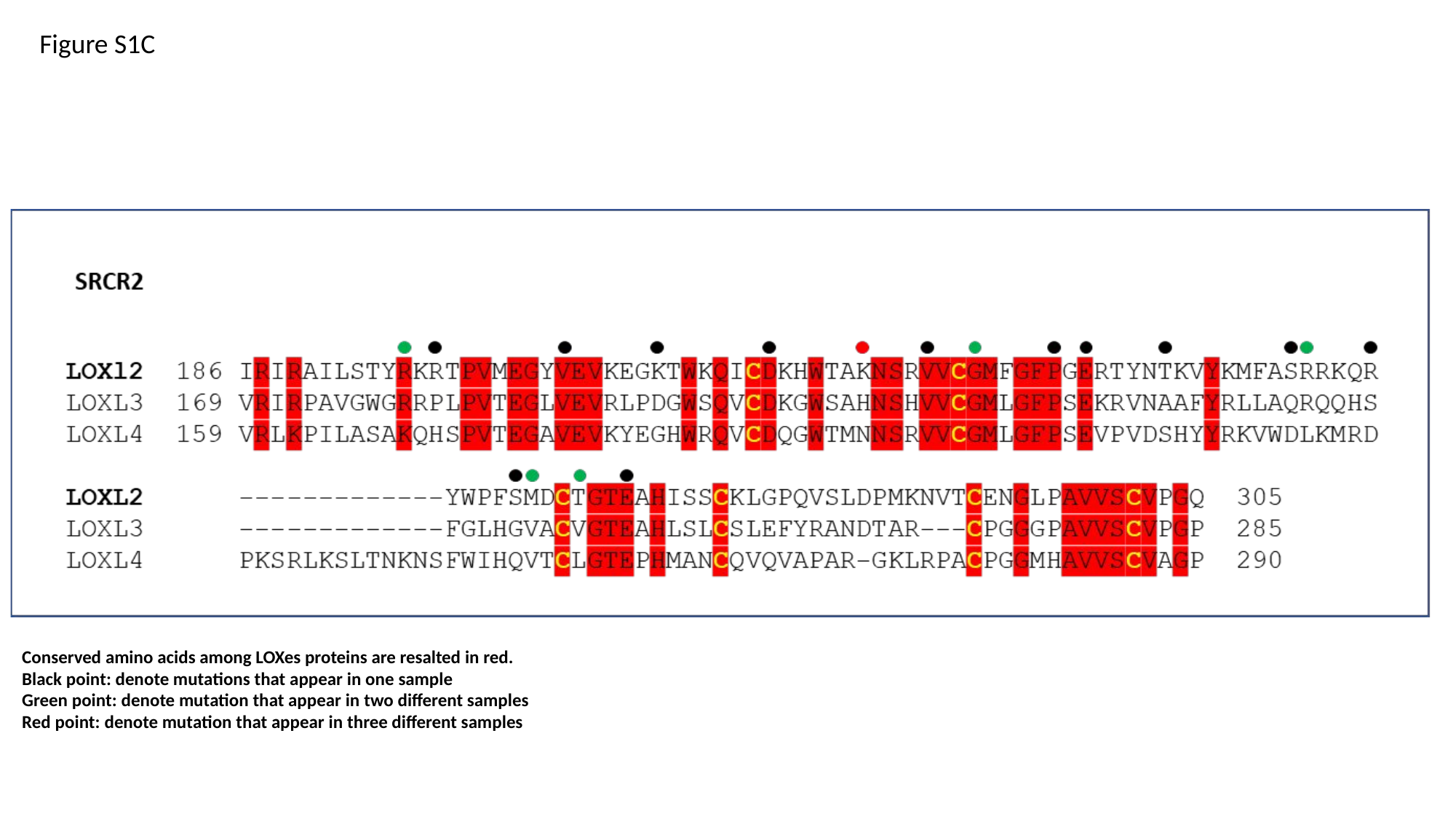

Figure S1C
Conserved amino acids among LOXes proteins are resalted in red.
Black point: denote mutations that appear in one sample
Green point: denote mutation that appear in two different samples
Red point: denote mutation that appear in three different samples

## Slide 4
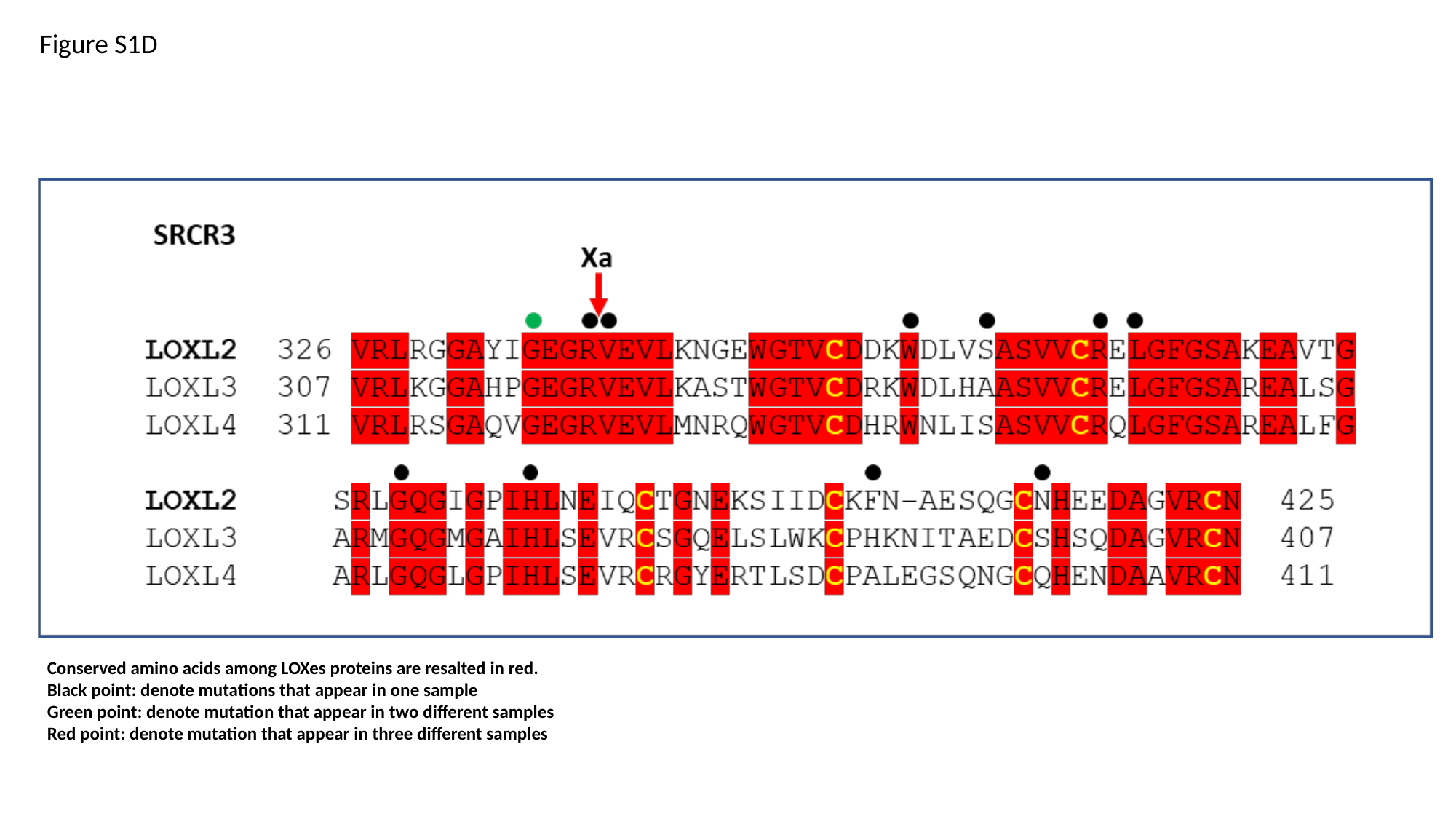

Figure S1D
Conserved amino acids among LOXes proteins are resalted in red.
Black point: denote mutations that appear in one sample
Green point: denote mutation that appear in two different samples
Red point: denote mutation that appear in three different samples

## Slide 5
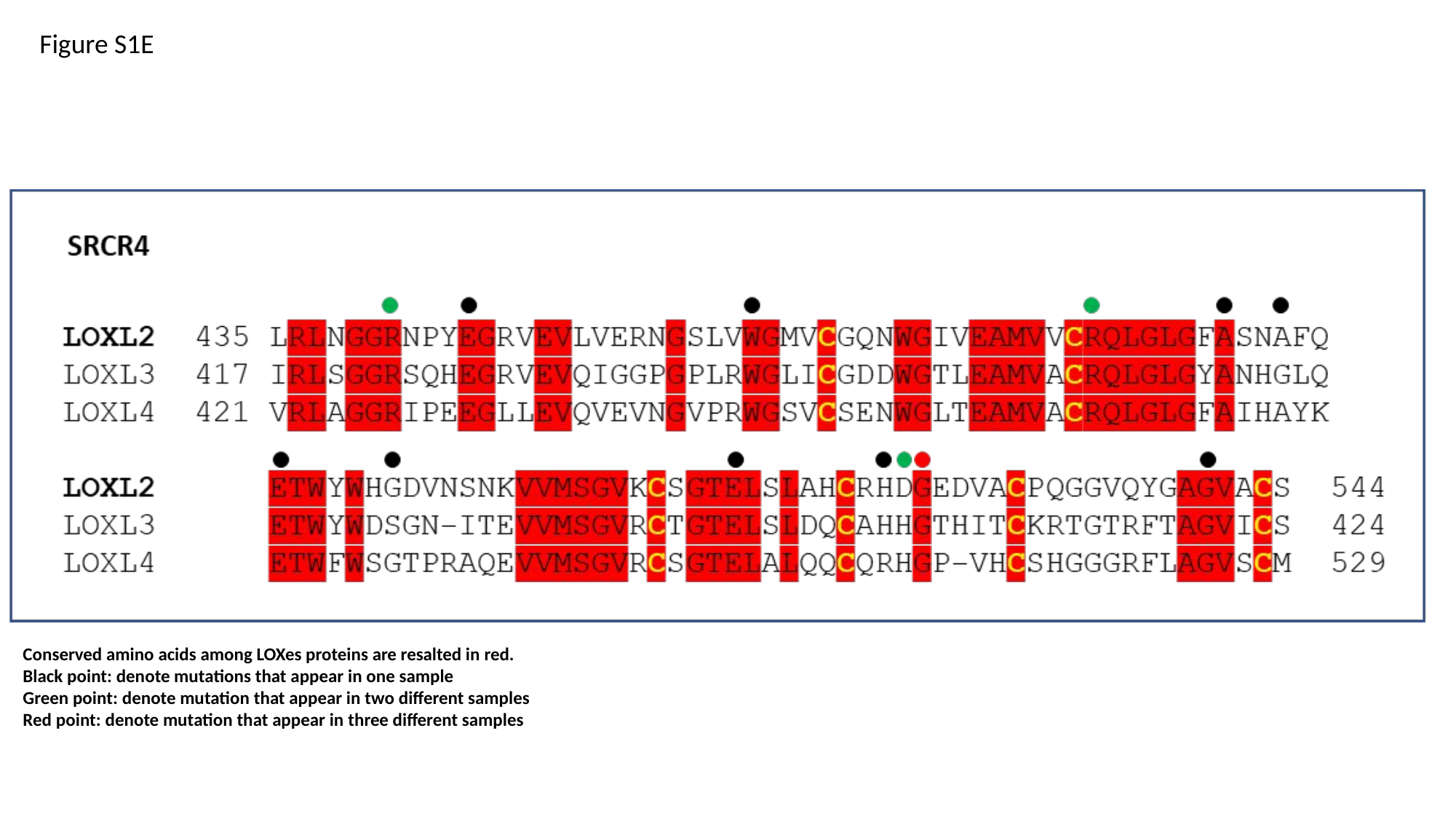

Figure S1E
Conserved amino acids among LOXes proteins are resalted in red.
Black point: denote mutations that appear in one sample
Green point: denote mutation that appear in two different samples
Red point: denote mutation that appear in three different samples

## Slide 6
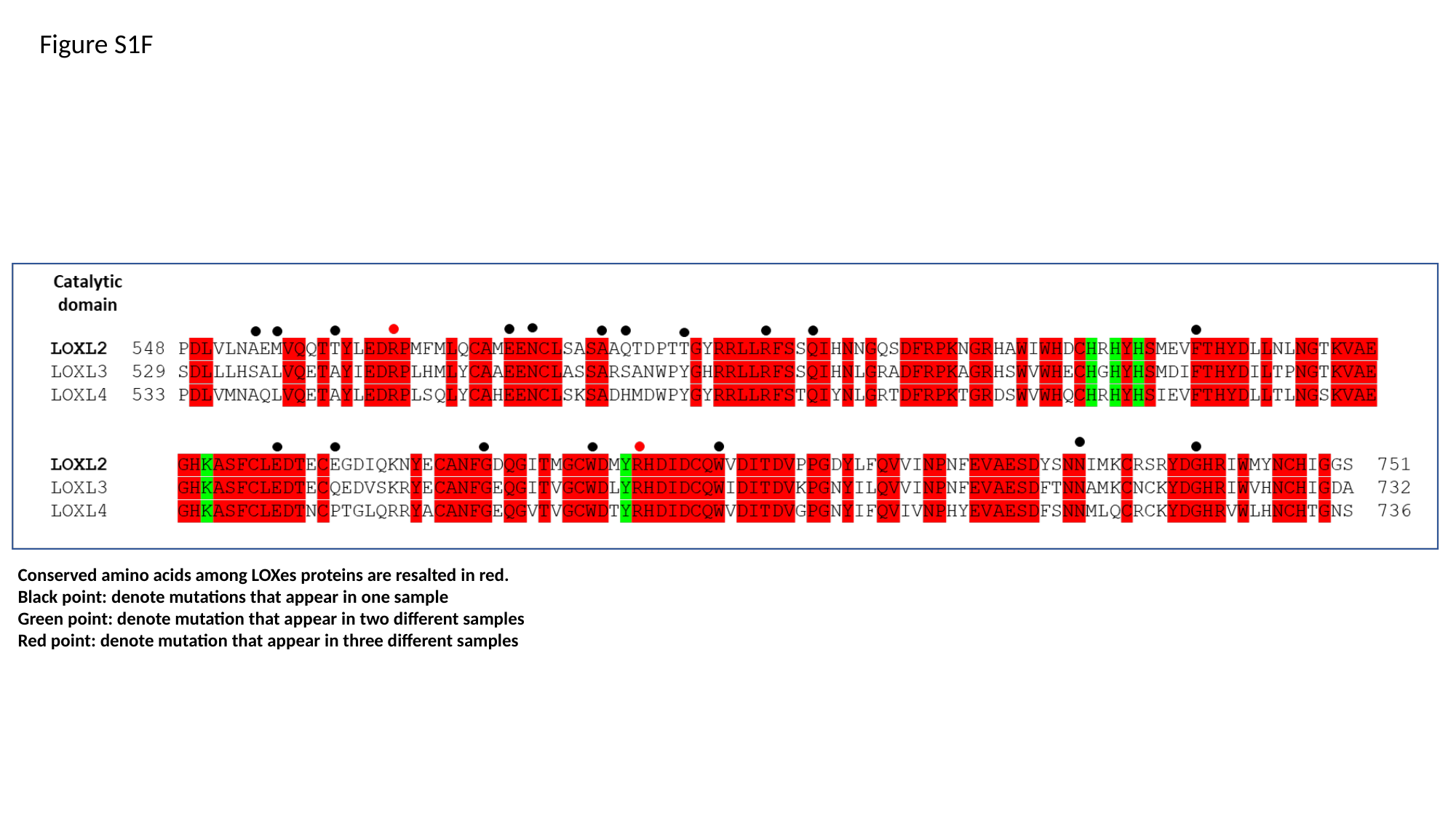

Figure S1F
Conserved amino acids among LOXes proteins are resalted in red.
Black point: denote mutations that appear in one sample
Green point: denote mutation that appear in two different samples
Red point: denote mutation that appear in three different samples

## Slide 7
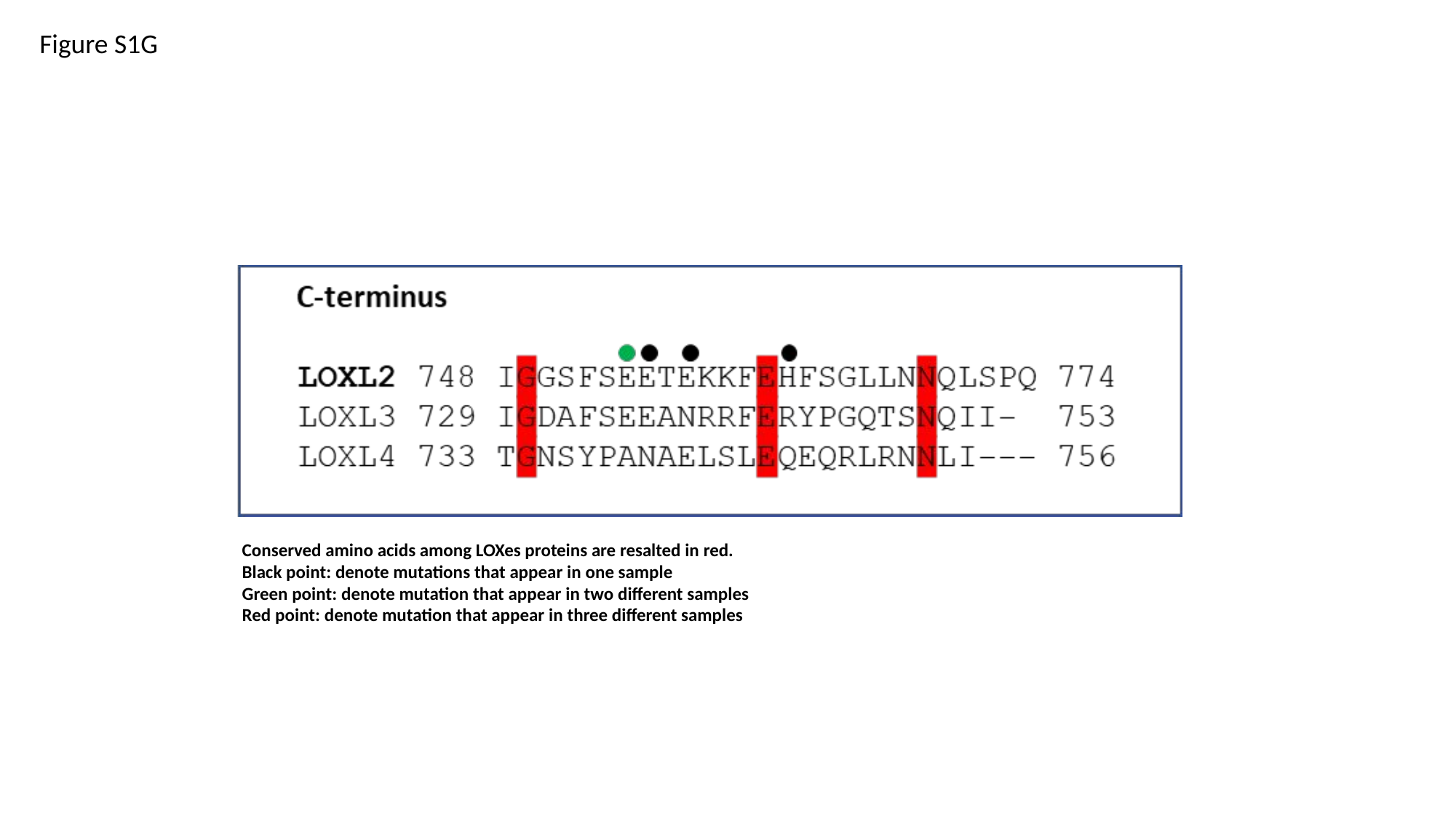

Figure S1G
Conserved amino acids among LOXes proteins are resalted in red.
Black point: denote mutations that appear in one sample
Green point: denote mutation that appear in two different samples
Red point: denote mutation that appear in three different samples
