# Supplementary material for: LOXL2 in Cancer: A Two-Decade Perspective
Source: Int J Mol Sci. 2023 Sep 21;24(18):14405. doi: 10.3390/ijms241814405 (PMC10532419; doi:10.3390/ijms241814405)
Supplement: Supplementary file 1 [file ijms-24-14405-s001.zip › Supplementary Materials captions.pdf]

Supplementary Materials

Supplementary Datasets

Dataset S1. LOXL2 in human cancers (an update of the last 5 years).

Dataset S2. Data from TCGA PanCancer Atlas showing copy number alterations and mutations found in LOXL2 locus.

Supplementary Figure Legend

Figure S1A–G. Localization of LOXL2 missense mutations in amino acid sequence alignment of LOXL2, LOXL3 and LOXL4 proteins. Conserved amino acids among LOXes proteins are resalted in red. Black point denote LOXL2 mutations that appear in only one PanCancer sample. Green point: denote mutations that appear in two different samples. Red point: denote mutation that appear in three different samples.

Supplementary Tables

Table S1. Small molecule inhibitors of LOXL2.
