# Supplementary material for: LOXL2 in Cancer: A Two-Decade Perspective
Source: Int J Mol Sci. 2023 Sep 21;24(18):14405. doi: 10.3390/ijms241814405 (PMC10532419; doi:10.3390/ijms241814405)
Supplement: Supplementary file 1 [file ijms-24-14405-s001.zip › Table S1.pdf]

Table S1. Small molecule inhibitors of LOXL2. ([a]nd: not determined).

Inhibitor	Structure	Specificity	IC50	pIC50	Reference
BAPN	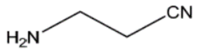	LOX/LOXL2	550 nM (LOX)<br>83 nM (LOX2)	[a]nd	Hutchinson JH, Rowbottom MW, Lonergan D, Darlington J, Prodanovich P, King CD, Evans JF, Bain G. Small Molecule Lysyl Oxidase-like 2 (LOXL2) Inhibitors: The Identification of an Inhibitor Selective for LOXL2 over LOX. ACS Med Chem Lett. 2017 Mar 1;8(4):423-427. doi: 10.1021/acsmedchemlett.7b00014.
PXS-S1A	the structure is not disclosed	LOX/LOXL2	nd	5.3 (LOX)<br>6.8 (LOX2)	Chang J, Lucas MC, Leonte LE, Garcia-Montolio M, Singh LB, Findlay AD, Deodhar M, Foot JS, Jarolimek W, Timpson P, Erler JT, Cox TR. Pre-clinical evaluation of small molecule LOXL2 inhibitors in breast cancer. Oncotarget. 2017 Apr 18;8(16):26066-26078. doi: 10.18632/oncotarget.15257.
PXS-S2A	the structure is not disclosed	LOX/LOXL2	nd	5.9 (LOX)<br>8.3 (LOX2)	Chang J, Lucas MC, Leonte LE, Garcia-Montolio M, Singh LB, Findlay AD, Deodhar M, Foot JS, Jarolimek W, Timpson P, Erler JT, Cox TR. Pre-clinical evaluation of small molecule LOXL2 inhibitors in breast cancer. Oncotarget. 2017 Apr 18;8(16):26066-26078. doi: 10.18632/oncotarget.15257.
PXS-5153A	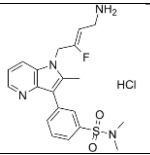	LOXL2/LOXL3	21 nM (LOXL2)<br>63 nM (LOXL3)	nd	Schilter H, Findlay AD, Perryman L, Yow TT, Moses J, Zahoor A, Turner CI, Deodhar M, Foot JS, Zhou W, Greco A, Joshi A, Rayner B, Townsend S, Buson A, Jarolimek W. The lysyl oxidase like 2/3 enzymatic inhibitor, PXS-5153A, reduces crosslinks and ameliorates fibrosis. J Cell Mol Med. 2019 Mar;23(3):1759-1770. doi: 10.1111/jcmm.14074. Epub 2018 Dec 9.
PAT-1251	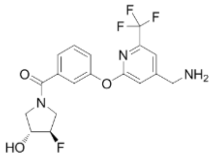	LOX/LOXL2	45 μM (LOX)<br>0.11 μM (LOXL2)	nd	Rowbottom MW, Bain G, Calderon I, Lasof T, Lonergan D, Lai A, Huang F, Darlington J, Prodanovich P, Santini AM, King CD, Goulet L, Shannon KE, Ma GL, Nguyen K, MacKenna DA, Evans JF, Hutchinson JH. Identification of 4-(Aminomethyl)-6-(trifluoromethyl)-2-(phenoxy)pyridine Derivatives as Potent, Selective, and Orally Efficacious Inhibitors of the Copper-Dependent Amine Oxidase, Lysyl Oxidase-Like 2 (LOXL2). J Med Chem. 2017 May 25;60(10):4403-4423. doi: 10.1021/acs.jmedchem.7b00345. Epub 2017 May 15. PMID: 28471663.
CCT365623	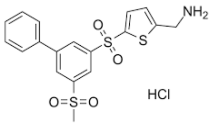	LOX/LOXL2	0.90 μM (LOX)<br>1.5 μM (LOXL2)	nd	Leung L, Niculescu-Duvaz D, Smithen D, Lopes F, Callens C, McLeary R, Saturno G, Davies L, Aljarah M, Brown M, Johnson L, Zambon A, Chambers T, Ménard D, Bayliss N, Knight R, Fish L, Lawrence R, Challinor M, Tang H, Marais R, Springer C. Anti-metastatic Inhibitors of Lysyl Oxidase (LOX): Design and Structure-Activity Relationships. J Med Chem. 2019 Jun 27;62(12):5863-5884. doi: 10.1021/acs.jmedchem.9b00335. Epub 2019 May 23.
Corilagin	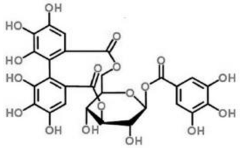	LOXL2	<1 μM	nd	Wei Y, Kim TJ, Peng DH, Duan D, Gibbons DL, Yamauchi M, Jackson JR, Le Saux CJ, Calhoun C, Peters J, Derynck R, Backes BJ, Chapman HA. Fibroblast-specific inhibition of TGF-β1 signaling attenuates lung and tumor fibrosis. J Clin Invest. 2017 Oct 2;127(10):3675-3688. doi: 10.1172/JCI94624.
Levoleucovorin	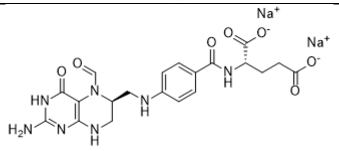	LOXL2	68.81 μM	nd	Deshpande H. Levoleucovorin inhibits LOXL2 (lysyl oxidase like-2) to control breast cancer proliferation: a repurposing approach. J Biomol Struct Dyn. 2023 Jun 20:1-10. doi: 10.1080/07391102.2023.2224894. Epub ahead of print.
